# Mechanistic aspects of IPTG (isopropylthio-β-galactoside) transport across the cytoplasmic membrane of *Escherichia coli*—a rate limiting step in the induction of recombinant protein expression

**DOI:** 10.1093/jimb/kuad034

**Published:** 2023-10-17

**Authors:** Rodrigo G Simas, Adalberto Pessoa Junior, Paul F Long

**Affiliations:** Faculty of Life Sciences & Medicine, King's College London, 150 Stamford Street, London SE1 9NH, UK; Faculdade de Ciências Farmacêuticas, Universidade de São Paulo, Av. Prof. Lineu Prestes, 580, B16, 05508-000 São Paulo, SP, Brazil; Faculty of Life Sciences & Medicine, King's College London, 150 Stamford Street, London SE1 9NH, UK; Faculdade de Ciências Farmacêuticas, Universidade de São Paulo, Av. Prof. Lineu Prestes, 580, B16, 05508-000 São Paulo, SP, Brazil; Faculty of Life Sciences & Medicine, King's College London, 150 Stamford Street, London SE1 9NH, UK; Faculdade de Ciências Farmacêuticas, Universidade de São Paulo, Av. Prof. Lineu Prestes, 580, B16, 05508-000 São Paulo, SP, Brazil

**Keywords:** IPTG, Simple diffusion, Active transport, *Escherichia coli*, lacY

## Abstract

Coupling transcription of a cloned gene to the lac operon with induction by isopropylthio-β-galactoside (IPTG) has been a favoured approach for recombinant protein expression using *Escherichia coli* as a heterologous host for more than six decades. Despite a wealth of experimental data gleaned over this period, a quantitative relationship between extracellular IPTG concentration and consequent levels of recombinant protein expression remains surprisingly elusive across a broad spectrum of experimental conditions. This is because gene expression under lac operon regulation is tightly correlated with intracellular IPTG concentration due to allosteric regulation of the lac repressor protein (lacY). An *in-silico* mathematical model established that uptake of IPTG across the cytoplasmic membrane of *E. coli* by simple diffusion was negligible. Conversely, lacY mediated active transport was a rapid process, taking only some seconds for internal and external IPTG concentrations to equalize. Optimizing *k*_cat_ and *K*_M_ parameters by targeted mutation of the galactoside binding site in lacY could be a future strategy to improve the performance of recombinant protein expression. For example, if *k*_cat_ were reduced whilst *K*_M_ was increased, active transport of IPTG across the cytoplasmic membrane would be reduced, thereby lessening the metabolic burden on the cell and expediating accumulation of recombinant protein. The computational model described herein is made freely available and is amenable to optimize recombinant protein expression in other heterologous hosts.

**One-Sentence Summary:**

A computational model made freely available to optimize recombinant protein expression in *Escherichia coli* other heterologous hosts.

## Graphical Abstract

**Graphical Abstract:**
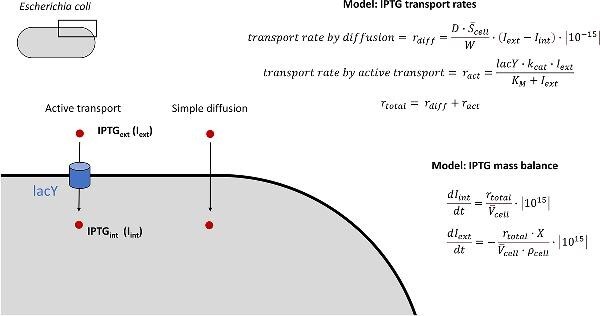
Model for IPTG diffusion in *Escherichia coli.*

## Introduction

Many different microorganisms and animal cell cultures are used as heterologous hosts to produce recombinant proteins for use as biopharmaceuticals. The first expression systems were derived from *Escherichia coli* strains, and these are still widely used today as cell factories to produce peptide therapeutics because of seminal knowledge accumulated over many decades, most notably pioneering studies elucidating mechanisms that govern gene expression first demonstrated by the *lac* operon.

Studies of the *lac* operon began in the 1950s with the discovery of ortho-nitrophenyl-β-galactoside (ONPG) as a synthetic substrate of β-galactosidase (Seidman & Link, 1950) and, the development of a simple and inexpensive method for quantification of β-galactosidase activity. In less than one decade after the invention of ONPG, the main components of the *lac* operon had been identified and, a detailed mechanism of gene regulation had been proposed (Pardee et al., 1959; Jacob & Monod, 1961; Jacob et al., 1964). Many fundamental concepts of molecular biology have subsequently been derived from these studies such as the nature of gene, the existence of operons and promoters, and mechanisms of gene repression and induction. In the *E. coli* wild type *lac* operon three genes are sequentially transcribed, encoding a β-galactosidase, a lactose permease (lacY), and a transacetylase (Müller-Hill, 1996). Lactose permease facilitates uptake of galactosides, particularly lactose, which is cleaved by β-galactosidase into galactose and allolactose (Kaback & Guan, 2019). The lac repressor binds to the operator sequence (Oehler et al., 1990), which is allosterically regulated by the natural inducer—allolactose, or analogs such as ONPG, methyl-1-thio-β-D-galactoside (TMG), isopropyl-1-thio-β-D-galactoside (IPTG), and β-D-galactopyranosyl 1-thio-β-D-galactopyranoside (TDG). (Kepes, 1960; Monod et al., 1965; Müller-Hill, 1996). The allosteric interaction of the inducer with the repressor modifies the affinity of the lac repressor protein for Deoxyribonucleic Acid (DNA) (Brewster et al., 2012; Razo-Mejia et al., 2018). In the absence of inducer–repressor binding, the repressor binds to the operator, obstructing Ribonucleic Acid (RNA) polymerase binding to the promoter (Müller-Hill, 1996). Conversely, binding of the inducer to the repressor leads to the release of the operator, allowing RNA polymerase to bind to the promoter. Thereafter, transcription is initiated upon binding of RNA polymerase to the promoter, followed by translation of the three proteins under the regulation of the lac operon.

To regulate operon expression, the inducer must first be transported from the extracellular space to the periplasm and then to the cytoplasm, with transport from the periplasm to the cytoplasm being the rate limiting step (Nichols, 2017). It is widely accepted that under customary physiological conditions, transport of small molecules into the cytoplasm occurs either by simple diffusion or by protein mediated transport (Viitanen et al., 1984; Carruthers, 1990; Lolkema et al., 1991; Kaback & Guan, 2019). With the advent of molecular biology, different parts of the lac operon have been modified to permit optimal expression of recombinant proteins by *E. coli*. The first adaptation was to the wild type promoter which was mutated so that the derivative, designated lacUV5, was no longer under cAMP regulation (Silverstone et al., 1970; Pastan & Adhya, 1976). Subsequently, the gene encoding RNA polymerase from phage T7 was cloned into the *E. coli* chromosome, so that this gene was also under the same regulatory mechanism as the lac operon (lacUV5 promoter/O1 operator) (Studier and Moffatt, 1986). Therefore, if a gene encoding a recombinant protein were cloned into a plasmid vector and then transformed into this modified *E. coli* strain, expression of the recombinant gene would also be regulated by the adapted lac operon (T7 promoter/O1 operator). In this way, both expression of the T7 RNA polymerase and recombinant protein would be modulated by the lac repressor, which in turn is regulated by the concentration of the inducer.

An unforeseen and unfortunate ramification was that expression of recombinant proteins increased the metabolic burden in this engineered *E. coli* strain, mainly due to the high transcription rate (Kosinski et al., 1992; Hoffmann & Rinas, 2004; Marbach & Bettenbrock, 2012; Li & Rinas, 2020). In response, numerous researchers have directed their efforts toward empirically exploring strategies to modulate transcription rates, for example, by either eliminating active transport of the inducer (Marbach & Bettenbrock, 2012; Binder et al., 2017), or using lower concentrations of the inducer (Turner et al., 2005; Marbach & Bettenbrock, 2012; Studier, 2014). Yet, a notable gap persists in rigorously applying modeling-based approaches to address this challenge. To achieve optimal recombinant protein expression using either scenario, it is fundamental to precisely understand the mechanism of inducer uptake, since the expression rate of the recombinant protein will be highly dependent upon the intracellular inducer concentration. With this aim, we have examined existing models for recombinant protein expression, including those developed by Noel et al. (2009), Calleja et al. (2014, 2016), Tran et al. (2015), Fernández-Castané et al. (2012), Santillán & Mackey (2004), Yildirim & Mackey (2003), and Ruiz et al. (2011). Surprisingly, the combination of simple diffusion and active transport of IPTG is rarely considered in these models. Common simplifications include assuming that in lacY^−^ cells, intracellular inducer concentration equals extracellular concentration, and that in lacY^+^ cells, diffusion transport is negligible. Furthermore, we have been unable to locate any biophysical model for IPTG diffusion into *E. coli* cells, a crucial element for developing a kinetic model for lacY^−^*E. coli* strains. Therefore, the objective of this study was to develop a kinetic model that would describe mechanisms and factors that govern the transport of inducers, specifically IPTG, across the cytoplasmic membrane of *E. coli*.

This was quantitatively attained by simulating simple diffusion and carrier mediated active transport processes, and then modeling the main variables affecting the uptake rate of IPTG by each process. We foresee that the model will provide valuable insights into the dynamics of IPTG uptake, enabling a more precise estimation of intracellular IPTG concentration. This concentration directly correlates with the allosteric regulation of the lac repressor, and subsequently, with transcription and translation processes.

## Methodology

The transport of IPTG across the cytoplasmic membrane of *E. coli* in a non-dividing culture was described using a kinetic model. In brief, the kinetic model computes the intracellular and extracellular IPTG concentrations over time, utilizing a mass balance principle. The approach accounts for two IPTG uptake pathways: simple diffusion and active transport, each represented by an algebraic equation describing the transport rate. Active transport is modeled using the Michalis–Menten equation, while a novel equation based on the Fick's law characterizes the simple diffusion of IPTG across the cytoplasmic membrane. Considerations for the modeling are detailed in the following sections and in the Supplementary Materials section. The model was implemented in Matlab (2021). Experimental data was collected retrospectively from relevant published literature.

### Mathematical Modeling

#### Assumptions and Simplifications

(i) There is one rate limiting step for transport into cells—One idiosyncrasy of transport across a series of membranes is that a composite permeability coefficient may be calculated from the coefficients of each membrane as resistances in parallel, according to Equation (1) (Nichols, 2017). Therefore, if the permeability coefficient of one membrane is much lower than the coefficients of the other membranes, then the composite coefficient is slightly lower than the lowest permeability coefficient, and it may be denominated as the rate limiting step for diffusion (Nichols, 2017). This is particularly true for diffusion of inducers into *E. coli* cells, which are delimited by an enveloped composed of three layers namely the cytoplasmic membrane, the peptidoglycan layer, and the outer membrane (Nanninga, 1998). In this case, diffusion across the cytoplasmic membrane, would be the rate limiting step (see Koch, 1990 and references therein).
  (1) \begin{equation*}\frac{1}{P} = \frac{1}{{{P}_1}} + \frac{1}{{{P}_2}} + \cdots + \frac{1}{{{P}_n}}.\end{equation*}
(ii) Small molecules may cross the cytoplasmic membrane by diffusion—The transport of small molecules across a membrane, such as a lipidic bilayer, may occur via simple diffusion since the membrane is classified as a fluid. The permeant size is considerably smaller than the membrane thickness, and the driving force for transport is the permeant concentration gradient across the membrane (Nichols, 2017). Although this classification is adequate, most studies on transport across membranes prefer the parameter “permeability coefficient” rather than “diffusion coefficient”. In fact, the permeability coefficient (*P*_m_), expressed in cm/s, is correlated with the diffusion coefficient (*D*), the partition coefficient between the permeant and the membrane (*K*), and the membrane width (*W*_m_) according to Equation (2) (Paula et al., 1996; Xiang & Anderson, 1997). In this study the term diffusion coefficient was preferred.
  (2) \begin{equation*}{P}_m = \frac{{KD}}{{{W}_m}}.\end{equation*}
(iii) Constant cell volume and preserved aspect-ratio of *E. coli* cells—Rod-shaped and coccoid bacterial species present a preserved aspect-ratio. Therefore, the surface area of a single cell is a function of its volume. For *E. coli*, the relation between cell volume (*V*, µm^3^) and surface area (*S*, µm^2^) follows the elegant form of Equation (3), as demonstrated by Ojkic et al. (2019).
  (3) \begin{equation*}S = 2\pi {V}^{2/3}.\end{equation*}

**Fig. 1.**
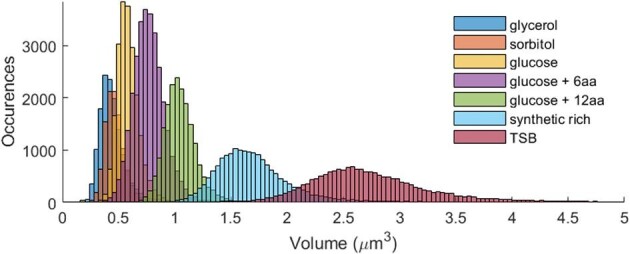
Distribution of newly generated cells growing in distinct cultivation media. The histogram was constructed from the original raw data from Taheri-Araghi et al. (2015), kindly provided for our study by the authors.

In any given cultivation, not all cells have the same cell volume, but it is expected that the volumes of the cells follow the same distribution. This has been experimentally demonstrated that the size of cells in each distinct growth condition followed a normal distribution, as depicted in Fig. 1 (Taheri-Araghi et al., 2015). However, for the purposes of this study the population will be described only by the average cell volume.

(iv) Transport via a permease is unidirectional in the absence of facilitated transport—The uptake of small molecules via permeases may happen via active or passive transport. Widdas (1952) proposed a facilitated transport mechanism for glucose across cell membranes, which was also demonstrated to be the case for inducer uptake by the lactose permease (Winkler & Wilson, 1966; Smirnova et al., 2011; Bosdriesz et al., 2018). Since active transport is only suppressed by metabolic inhibitors (“metabolic poisons”, see Winkler and Wilson, 1966) and the expected uptake rate via active transport is much higher than the facilitated transport rate (Bosdriesz et al., 2018), we decided not to consider the effect of facilitated transport in this study.
(v) Constant number of transport proteins per area—The quantity of protein molecules in a cell is known to be subject to significant variations and is dependent on various factors such as cultivation conditions, cellular physiological state and the heterogeneous nature of the culture (Taniguchi et al., 2010; Li & Xie, 2011). This is especially relevant in the case of the lactose permease in recombinant *E. coli* strains grown under induced conditions, as protein expression is controlled by the *lac* operon, which is regulated by the same inducer as the recombinant protein. A measurement of protein copy number is typically performed by determining molar concentration. However, to simplify our model and to investigate the effect of cell volume, we have chosen to express the number of lacY molecules per unit of cell surface (lacY molecules·μm^−3^). The total number of molecules was then calculated by multiplying this value by the average cell surface area.
(vi) Low cell concentration—So as to maintain a consistent extracellular inducer concentration throughout the simulations, a low cell concentration of 0.01 g/L was considered.
(vii) Constant number of cells—Exclusion of cell division in the experimental design was intentional, as this would have introduced a dilution factor that might complicate visual and analytical interpretation of the results.
(viii) Constant temperature—Temperature is recognized to have a positive influence on diverse physiological parameters, such as diffusion coefficient, membrane composition, transport rate, cell size, and cell growth rate. Nevertheless, for the sake of model simplification and facilitating comparisons with existing literature data, we treated temperature as a constant factor.

#### Mass Balance

The model was designed to evaluate IPTG concentration in a culture of non-dividing *E. coli* cells. For the intracellular IPTG concentration, the boundary of the system was delimited by the cytoplasmic cell membrane, which was the rate limiting step for diffusion of IPTG into the cells. For the extracellular IPTG concentration, the boundary was the limit of the reaction volume. Our interest was to study the transient state of IPTG transport by means of a kinetic model. To simplify the modeling, the following assumptions were considered: (a) constant number of inducer transporters in each cell, (b) constant average cell volume, (c) constant diffusion coefficient of IPTG, (d) constant reaction volume, (e) low cell concentration, (f) constant number of cells (no division), (g) diffusion across the cytoplasmic membrane is the rate limiting step, (h) no carrier mediated efflux of IPTG, and (i) no carrier mediated facilitated transport of the IPTG. Hence, the cytoplasmatic or intracellular IPTG concentration (*I_i_*_nt_) and extracellular IPTG concentration (*I*_ext_) were calculated by mass balance (Equations 4 and 5), as a function of the total IPTG transport rate into the cells (*r*_total_), average cell volume (${\bar{V}}_{{\mathrm{cell}}}$), cell concentration (*X*) and cell density (ρ_cell_). The detailed calculation procedure is presented in the Supplementary Material.


(4) \begin{equation*}\frac{{d{I}_{int}}}{{dt}} = \frac{{{r}_{total}}}{{{{\bar{V}}}_{cell}}} \cdot \left| {{{10}}^{15}} \right|,\end{equation*}

(5) \begin{equation*}\frac{{d{I}_{ext}}}{{dt}} = - \frac{{{r}_{total} \cdot X}}{{{{\bar{V}}}_{cell} \cdot {\rho }_{cell}}} \cdot \left| {{{10}}^{15}} \right|.\end{equation*}

Simple diffusion and active transport are independent processes and the individual contribution of each to the total transport rate are additive (Maloney & Hastings Wilson, 1973), as per Equation (6), where *r*_total_ is a combination of simple diffusion (*r*_diff_) and carrier mediated active transport (*r*_act_).


(6) \begin{equation*}{{{r}}}_{{{total}}} = {\mathrm{\ }}{{{r}}}_{{{diff}}} + {{{r}}}_{{{act}}}.\end{equation*}

#### Simple Diffusion

An expression for the rate of simple diffusion per cell (*r*_diff_) was built based on Fick's law, as detailed in the Supplementary Material. *r*_diff_ is a function of the diffusion coefficient of IPTG at the cytoplasmic cell membrane (*D*), the average surface area of one cell (${\bar{S}}_{{\mathrm{cell}}}$), the extracellular IPTG concentration (*I*_ext_), the intracellular IPTG concentration (*I*_int_), and the average cytoplasmic membrane width (*W*) according to Equation (7). Due to the preservation of aspect-ratio in rod-shaped and coccoid bacterial species (Ojkic et al., 2019), surface area was substituted by an equivalent expression, in which the cell volume is the independent variable (Equation 8).


(7) \begin{equation*}{r}_{diff} = \frac{{D \cdot {{\bar{S}}}_{cell}}}{W} \cdot \left( {{I}_{ext} - {I}_{int}} \right) \cdot \left| {{{10}}^{ - 15}} \right|,\end{equation*}

(8) \begin{equation*}{\bar{S}}_{cell} = 2\pi {\bar{V}}_{cell}^{2/3}.\end{equation*}

#### Active Transport

Active transport of IPTG across the cytoplasmic cell membrane of *E. coli* via lacY follows the Michalis–Menten equation (Rickenberg et al., 1956), where *r*_act_ is the rate of IPTG uptake per cell (Equation 9), *I*_ext_ is the extracellular IPTG concentration, *k*_cat_ is the turnover number, *K*_M_ is the dissociation constant of the complex IPTG-lacY, and lacY is the average number of lactose permease molecule per cell (Equation 10); which was calculated from the average number of lactose permease molecules per cell surface area (*N*_lacY_), the average cell surface area (${\bar{S}}_{{\mathrm{cell}}}$) and the Avogadro number (*N*_A_). The maximum uptake rate per cell volume (*v*_max_) was calculated according to the Equation (11).


(9) \begin{equation*}{r}_{act} = \frac{{lacY \cdot {k}_{cat} \cdot {I}_{ext}}}{{{K}_M + {I}_{ext}}},\end{equation*}

(10) \begin{equation*}lacY = \frac{{{N}_{lacY} \cdot {{\bar{S}}}_{cell}}}{{{N}_A}},\end{equation*}

(11) \begin{equation*}{v}_{max} = \frac{{lacY*{k}_{cat}}}{{{{\bar{V}}}_{cell}}}*\left| {6.0 \cdot {{10}}^{19}} \right|.\end{equation*}

#### List of Variables

A list of all variables used in the model is presented in Table 1.

**Table 1.** List of variables used in the model for simple and facilitated diffusion

Variable	Description	Unit
*I_ext_*	Extracellular IPTG concentration	mol L^−1^
*I_int_*	Intracellular IPTG concentration	mol L^−1^
*D*	Diffusion coefficient of IPTG across the cytoplasmic membrane	μm^2^ s^−1^
*dI*	Variation of IPTG concentration across the cytoplasmic membrane	mol μm^−3^
*lacY*	Quantity of lactose permease per cell	mol cell^−1^
*k_cat_*	Turnover number of IPTG uptake by lacY	s^−1^
*K_M_*	Michaelis constant for IPTG uptake by lacY	mol L^−1^
*N_A_*	Avogadro number (6.02214076·10^23^ mol^−1^)	mol^−1^
*N_lacY_*	Lactose permease copy number per cell surface area	molecules µm^−2^
*r_diff_*	IPTG uptake rate by simple diffusion	mol s^−1^ cell^−1^
*r_act_*	IPTG uptake rate by active transport	mol s^−1^ cell^−1^
*r_total_*	Total IPTG uptake rate	mol s^−1^ cell^−1^
${{\boldsymbol{\bar{S}}}}_{{\boldsymbol{cell}}}$	Average surface area of one cell	µm^2^
*t*	time	s
${{\boldsymbol{\bar{V}}}}_{{\boldsymbol{cell}}}$	Average volume of one cell	µm^3^
*v_max_*	Maximum uptake rate per cell volume	μmol min^−1^ cm^−3^
*X*	*E. coli* cell concentration	g L^−1^
*W*	Average width of cytoplasmic membrane	μm
*ρ_cell_*	Cell density of wet *E. coli* cells	g L^−1^

#### Diffusion Time

Diffusion time (*t*_d_) was defined as the time needed for *I*_int_ to achieve a certain fraction of *I*_ext_, defined as a threshold (θ_d_) multiplied by *I*_int_. We considered θ_d_ as 90%.

### Programming—ODEs

The model was implemented and solved in Matlab R2021a^®^. The ordinary differential equations were solved using the method *ode15s*. Sensitivity analysis was carried out in Simulink using the method *ode.analyze*, in which the cost function was defined as the diffusion time, as defined previously.

### Data From the Literature

The lactose permease of *E. coli* acts as the primary transporter for lactose into the cells, with the capability of transporting several other galactosides such as ONPG, TMG, and IPTG. To estimate the parameters for IPTG transport, retrospective experimental data of lacY substrate available in the scientific literature were used in this study (Table 2).

**Table 2.** Resume of kinetic parameters for lactose permease mediated transport across the *E. coli* cytoplasmic membrane

Parameter		Value	Reference
Affinity (*K*_m_) of external galactoside and lacY (influx)	Lactose	200 μM	(Robertson et al., 1980)
		260 ± 30 μM	(Lancaster et al., 1975)
		280 ± 50 μM	(Huber et al., 1980)
		400 μM	(Dekel and Alon, 2005)
		470 ± 130 μM	(Viitanen et al., 1984)
		600–1000 μM	(Winkler and Wilson, 1966)
		2.5 mM	(Lolkema et al., 1991)
	NPG	500–1300 μM	(Winkler and Wilson, 1966)
	TMG	500 μM	(Kepes, 1960)
		700 μM	(Ozbudak et al., 2004)
		800 μM	(Maloney & Hastings Wilson, 1973)
	TDG	20 μM	(Kepes, 1960)
	TPG	250 μM	(Kepes, 1960)
Turnover number of lacY		16–21 s^−1^	(Viitanen et al., 1984)
		21 ± 3.6 s^−1^	(Smirnova et al., 2011)
Cytoplasmic membrane thickness		37.5 Å	(Mitra et al., 2004)

## Results

To evaluate the model, simple diffusion and active transport were analyzed separately, first by the one-variable-at-a-time (OVAT) strategy followed by a sensitivity analysis. Lastly, the combined effects of simple diffusion and active transport were considered. For all simulations, the initial extracellular and intracellular concentrations of IPTG were 1.0 mM and 0.0 mM, respectively.

### Simple Diffusion

**Fig. 2.**
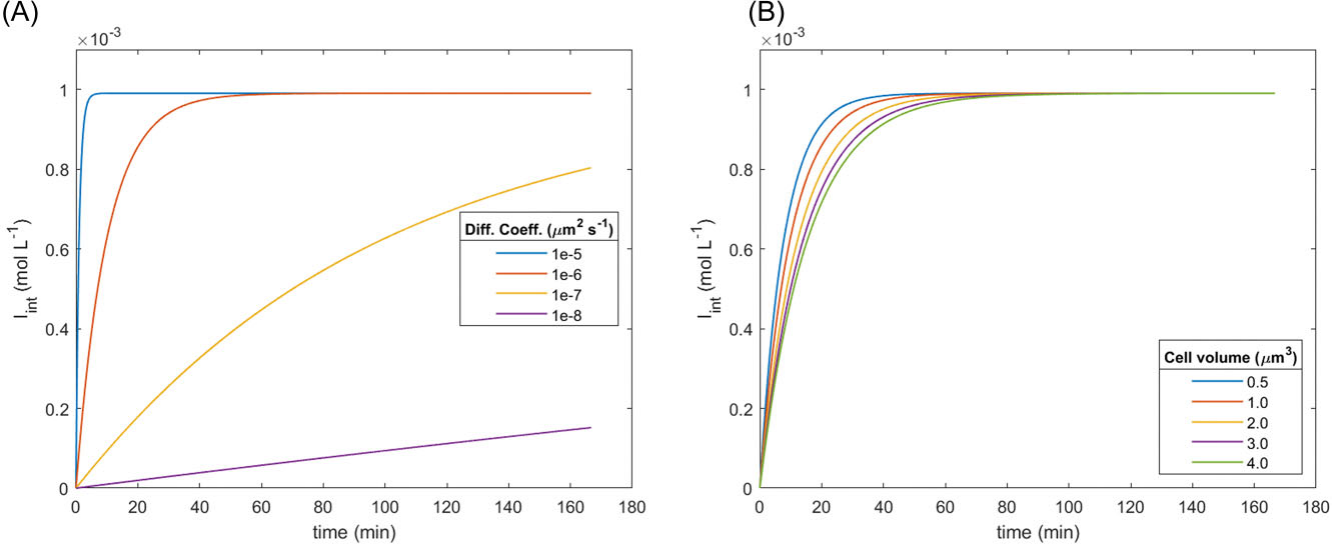
Effect of diffusion coefficient and average cell volume on simple diffusion of IPTG. Analysis using an OVAT strategy to evaluate the effect of (A) Diffusion coefficient = 1 · 10^−6^ μm^2^ s^−1^, on simple diffusion of IPTG and (B) Average cell volume constant = 1.044 μm^3^.

The effect of two variables—cell volume and diffusion coefficient—on *I*_int_ were analysed by the OVAT strategy (Fig. 2). For the simulated conditions, rapid equilibration (diffusion time <5 min) was achieved when the diffusion coefficient (*D*) was around 10^−5^ μm^2^ s^−1^ and a diffusion time lower than 1 hr was observed when a value for *D* was around 10^−6^ μm^2^ s^−1^ (Fig. 2A). Conversely, the time for diffusion when *D* was lower than 10^−7^ μm^2^ s^−1^ increased to approximately 4 hr.

The impact of cell volume on diffusion time was less pronounced compared to the effect of the diffusion coefficient. Despite a wide range of cell volumes being selected based upon the experimental data from Taheri-Araghi et al. (2015), the calculated diffusion time was within the range of 10–60 min (as seen in Fig. 2B). For a given cultivation condition with a defined maximum specific growth rate, the cell volume distribution was expected to exhibit a limited range. For instance, Taheri-Araghi et al. (2015) verified that the cell volume of *E. coli* cells in a culture with a maximum growth rate of 1.605 ± 0.496 h^−1^ was 1.044 ± 0.153 μm^3^.

**Fig. 3.**
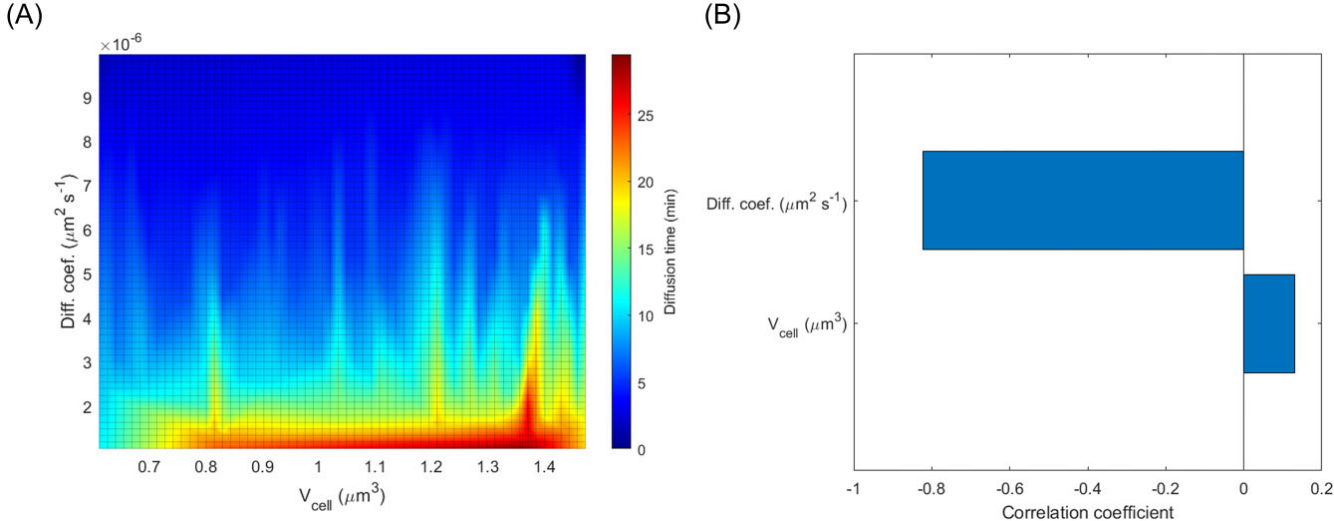
Sensitivity analysis of active transport of IPTG considering the combined effect of diffusion coefficient and cell volume. The model was evaluated for 100 samples, with the (A) diffusion coefficient evaluated in the range of 1 · 10^−6^– 1 · 10^−5^ μm^2^ s^−1^ and (B) cell volumes evaluated from 0.6 to 1.5 μm^3^.

A sensitivity analysis was next conducted to evaluate the relative impact of variation in diffusion coefficients and cell volumes on diffusion time. The analysis was performed *in silico* and comprised 100 experiments, each incorporating a randomly selected diffusion coefficient ranging from 1·10^−6^ to 1·10^−5^ μm^2^ s^−1^, and a randomly selected cell volume between 0.6 and 1.5 μm^3^. The results of this analysis indicated that the diffusion coefficient has a significantly negative effect on the diffusion time (Fig. 3A), while cell volume had only a modest positive effect (Fig. 3B). This suggested that higher values of *D* resulted in lower diffusion times, and that larger cell volumes only slightly increased the diffusion time.

### Active Transport

**Fig. 4.**
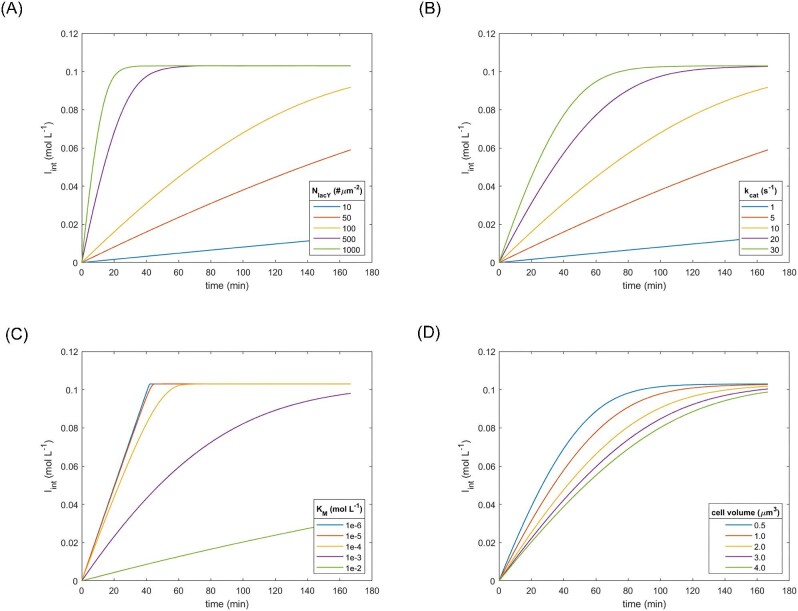
OVAT modeling of IPTG uptake by an active transport mechanisms (A) effect of *N*_lacY_, (B) Effect of *k*_cat_, (C) effect of *K*_M_, (D) effect of ${\bar{V}}_{{\mathrm{cell}}}$.

**Fig. 5.**
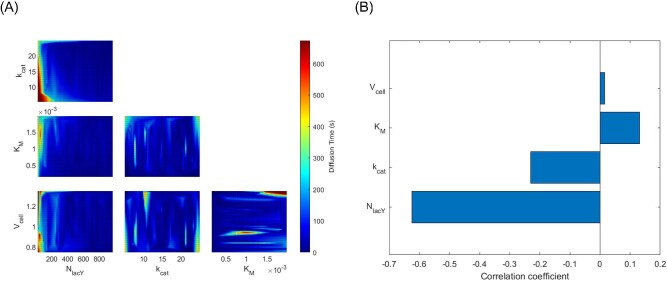
Sensitivity analysis of active transport of IPTG when the combined effects of *N*_lacY_, *k*_cat_, *K*_M_, and ${\bar{V}}_{{\mathrm{cell}}}$ were considered. *N*_lacY_ was evaluated in the range from 10 to 1000 molecules·μm^−2^, *k*_cat_ was evaluated from 5.0 to 25.0 s^−1^, *K*_M_ was evaluated from 0.1 to 2.0 mM, and ${\bar{V}}_{{\mathrm{cell}}}$  was  evaluated from 0.738 to 1.350 μm^3^.

The uptake rate of IPTG due to active transport was calculated using Equations (9), (10), and (11), which included four independent variables (*N*_lacY_, *k*_cat_, *K*_M_, ${\bar{V}}_{{\mathrm{cell}}}$). The effect of each variable was first evaluated by an OVAT (Fig. 4), followed by a sensitivity analysis (Fig. 5). For the OVAT analysis, the “standard values” of the independent variables were chosen based upon the data from Table 2, as follows: *N*_lacY_–200 molecules·μm^−2^, *k*_cat_–20.0 s^−1^, *K*_M_–5 · 10^−4^ M, and ${\bar{V}}_{{\mathrm{cell}}}$–1.044 μm^3^. The constants were: *X*–0.01 g·L^−1^, ρ_cell_–1.03 g·L^−1^, *D*–0.0 μm^2^·s^−1^, and *W*–3.75 · 10^−3^ μm.

In all cases presented in Fig. 4, the intracellular IPTG concentration rapidly overcame the initial extracellular IPTG concentration of 1 mM, because of active transport. Some conditions indicated that the equilibrium was reached at around 100 mM, which corresponded to a concentration 100× higher than the initial extracellular IPTG concentration. For the uptake of TMG by *E. coli*, a 100× concentration factor was also reported by Rickenberg et al. (1956). The number of enzymes per surface area (Fig. 4A) greatly affected the kinetic profile of IPTG uptake. While 10 lacY molecules per μm^3^ seemed to be insufficient to quickly reach equilibrium, 1000 molecules per μm^3^ lead to a faster response, in which the equilibrium was reached after around 30 min. In this case, the intracellular concentration of IPTG was the same as the extracellular concentration after just 7 s. The effect of turnover number presented a similar profile (Fig. 4B). The shortest equilibrium time was around 100 min with a *k*_cat_ equal to 30 s^−1^. Smaller turnover numbers lead to increasingly longer equilibration times. In the ranges studied, when the *K*_M_ was lower than 10^−4^ mol·L^−1^ the equilibration times were less than 1 hr (Fig. 4C). When the *K*_M_ was 10^−3^ mol·L^−1^, the equilibration time was longer than 3 hr, even though the intracellular IPTG concentration was the same as the extracellular concentration after around 4 min. The smallest impact was observed when cell volume was considered as the variable (Fig. 4D). Even when a wide range of values were considered, the equilibration time only varied from 60 min to around 180 min.

A sensitivity analysis was conducted to determine the combined effect of the four variables, *N*_lacY_, *k*_cat_, *K*_M_, and ${\bar{V}}_{{\mathrm{cell}}}$. One hundred *in silico* experiments were performed, each incorporating random values within predefined ranges for each variable. The range for *N*_lacY_ was set between 10 and 1000 molecules·μm^−2^, for *k*_cat_ between 5.0 and 25.0 s^−1^, for *K*_M_ between 0.1 and 2.0 mM, and for ${\bar{V}}_{{\mathrm{cell}}}$ between 0.738 and 1.350 μm^3^. The results of the simulations demonstrated that most of the diffusion times were below 200 s (Fig. 5A) and that *N_lacY_* was the variable that had the most significant impact upon the diffusion time, whereas cell volume only had a negligible effect (Fig. 5B).

### Combined Transport

**Fig. 6.**
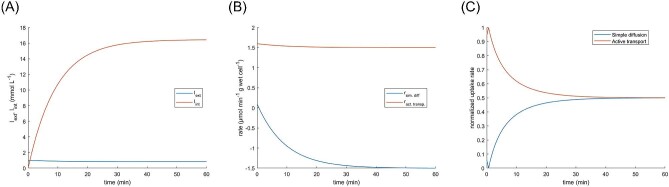
Simulation of combined simple diffusion and active transport of IPTG across the *E. coli* cell membrane.

A simulation was performed to evaluate the relative contribution of simple diffusion and active transport on the rate of IPTG uptake. The parameters were estimated based upon a review of the literature and prior simulations, as follows: *N*_lacY_ = 200 molecules·μm^−2^, *k*_cat_ = 20 s^−1^, *K*_M_ = 5 · 10^−4^ M, ${\bar{V}}_{{\mathrm{cell}}}$ = 1.044 μm^3^, *D* = 1 · 10^−6^ μm^2^·s^−1^, and membrane width = 3.75 · 10^−3^ μm (Fig. 6). Intracellular IPTG concentration was determined to be equal to the extracellular concentration after 36 s (Fig. 6A). The initial rate of simple diffusion was 0.0962 μmol min^−1^ g wet cell^−1^ (Fig. 6B), which agreed with experimental observations between 0.002 and 0.2 μmol min^−1^ g wet cell^−1^ for lactose and NPG (Winkler & Wilson, 1966). On the other hand, the calculated maximum rate of active transport (lacY · *k*_cat_) was 2.40 μmol min^−1^ g wet cell^−1^, which was around one order of magnitude lower than values reported in the literature of 15–106 μmol min^−1^ g wet cell^−1^ (Winkler & Wilson, 1966; Maloney & Hastings Wilson, 1973). After approximately 40 min, the system reached equilibrium with a constant IPTG intracellular concentration of 16.4 mM (Fig. 6A and C).

**Fig. 7.**
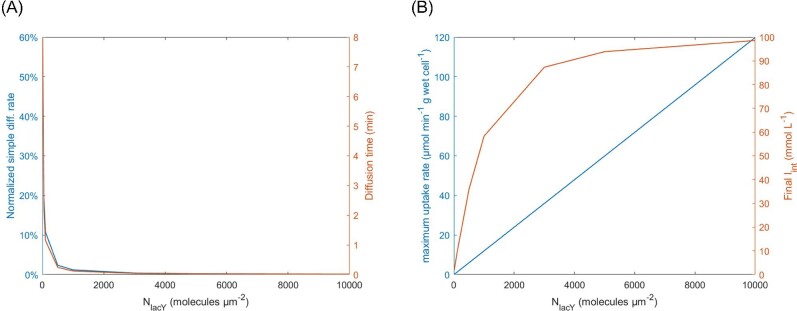
Effect of the number of transporters per area (*N_lacY_*) on diffusion time (A) on the normalized contribution of simple diffusion to total transport rate and (B) on the maximum IPTG uptake rate and on the final intracellular IPTG concentration.

Another important aspect was to consider the contribution of simple diffusion on the rate of IPTG uptake. As the rate of active transport is highly dependent on the number of enzymes (as discussed earlier), it was useful to plot the contribution of simple diffusion to the total IPTG uptake rate as function of *N*_lacY_ (Fig. 7). When *N*_lacY_ was equal to 10 molecules per μm^2^, simple diffusion accounted for 54.6 % of the total IPTG transport rate (Fig. 7A). While when *N*_lacY_ was equal to 1000 and 10 000 molecules per μm^2^, the contribution of simple diffusion plunged to 1.2 % and 0.1 %, respectively (Fig. 7B). From Fig. 7B, the equilibrium concentration of intracellular IPTG became limited with increasing number of enzymes per cell surface area. The difference between the concentration limit and the equilibrium concentration was proportional to the relative contribution of the rate of simple diffusion (Fig. 7A and B).

**Fig. 8.**
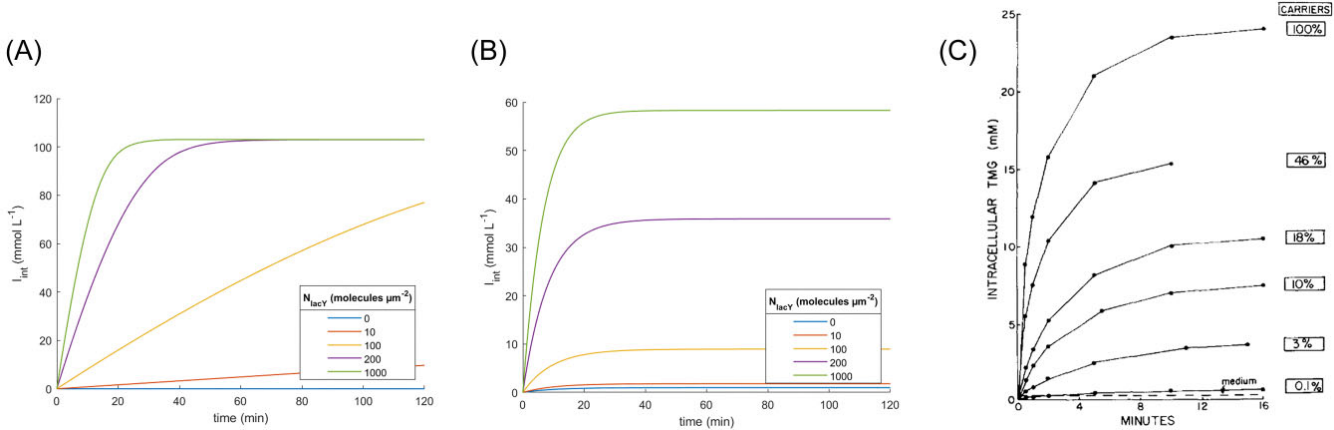
Kinetic profiles of intracellular IPTG concentrations for different numbers of carriers. (A) Active transport without simple diffusion, (B) combination of active transport and simple diffusion, and (C) experimental data from Maloney & Hastings Wilson, (1973) for the transport of TMG in *E. coli*.

**Fig. 9.**
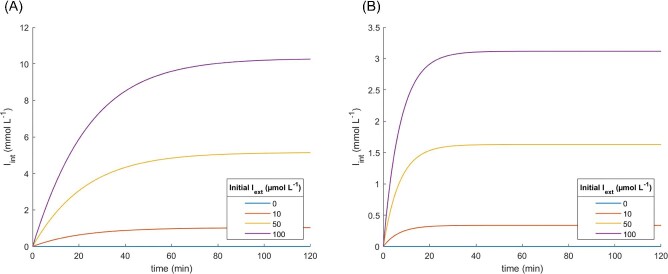
Kinetic profile of intracellular IPTG concentration for initial extracellular IPTG concentration ranging from 0 to 100 μM. Simulation parameters: *k*_cat_ = 20 s^−1^, *K*_M_ = 5 · 10^−4^ M, ${\bar{V}}_{{\mathrm{cell}}}$ = 1.044 μm^3^, and *N*_lacY_ = 200 molecules·μm^−2^. (A) Active transport without simple diffusion, *D* = 0 μm^2^·s^−1^ and (B) combination of active transport and simple diffusion, *D* = 1 · 10^−6^ μm^2^·s^−1^.

The contribution of simple diffusion was further investigated in subsequent simulations (Figs. 8A and B and 9A and B). First, the *I*_int_ kinetic profile in presence and absence of simple diffusion was evaluated for several values of *N*_lacY_. Consistent with previous simulations, the absence of simple diffusion resulted in a 100-fold IPTG concentration factor, which was found to be independent of the number of lacY molecules (Fig. 8A). Incorporating simple diffusion (*D* = 1 · 10^−6^ μm^2^·s^−1^), the IPTG concentration factor displayed a clear correlation with the number of lactose permease molecules (Fig. 8B). This finding supported the experimental results reported by Maloney & Hastings Wilson, (1973) (Fig. 8C). Conversely, varying *I*_ext_ demonstrated that *I*_int_ was correlated with *I*_ext_, irrespective of the presence or absence of simple diffusion (Fig. 9A and B).

## Discussion

Models describing the active transport of inducer by lactose permease have been extensively explored since the identification of this permease. The consensus within the field is that the rate of inducer uptake via active transport adheres to a Michaelis–Menten equation, with the rate being a function of the extracellular inducer concentration (Rickenberg et al., 1956; Kepes, 1960). It was only several years later that the role of simple diffusion was incorporated into a model by Maloney & Hastings Wilson (1973). This pioneering work suggested that, in addition to active transport, inducer transport is also enabled by diffusion. Subsequently, various models were formulated, including those by Noel et al. (2009), Tran et al. (2015), and Calleja et al. (2014).

Maloney & Hastings Wilson (1973) and Noel et al. (2009) theorized that IPTG influx primarily occurs through active transport, while diffusion is predominantly attributed to IPTG efflux. In such instances, the diffusion rate (*r*) is modelled using first-order kinetics, which is a function of the intracellular IPTG concentration (IPTG_int_) and the diffusion constant (*k*): *r* = *k* · *IPTG_int_*.

In contrast, Tran et al. (2015) formulated a model exclusively based on simple diffusion, omitting active transport mechanisms. This distinctive approach incorporates bidirectional inducer diffusion, setting it apart from earlier models. Like its predecessors, this model employs first-order kinetics to describe diffusion, although lacking direct physical significance. Using a similar approach, Calleja et al. (2014) devised a model specifically tailored for lacY-negative strains, focusing solely on simple diffusion. In this particular model, IPTG transport is characterized as a first-order reaction, being the transport rate proportional to the difference between intracellular and extracellular inducer concentrations. Consistent with preceding models, the diffusion constant is empirically derived, and its connection to physical parameters remains absent.

Our model builds upon the mentioned earlier approaches and comprises both active transport and diffusion. Specifically, we incorporate bidirectional simple diffusion, adhering to Fick's law, with the driving force being the difference in IPTG concentration between the extracellular and intracellular compartments. This diffusion rate is quantified as a function of biophysical parameters, such as the diffusion coefficient of the inducer in the cytoplasmic membrane and cellular dimensions. Further elaboration on the quantitative data generated by our model, along with a comprehensive comparison to literature data, will be provided in the subsequent sections.

### Simple Diffusion

When a cell volume of 1.044 μm^3^ was considered, short equilibration times (< 1 hr) were reached only when the diffusion coefficient of IPTG across the cytoplasmic membrane was greater than 1 · 10^−6^ μm^2^ s^−1^ (Fig. 2B). In the face of experimental claims that IPTG uptake is a quick process and, quantitative data that suggested diffusion times ranged from 1 to 104 min (Winkler & Wilson, 1966; Tran et al., 2015), it was not implausible to consider that the diffusion coefficient of IPTG across the cytoplasmic membrane of *E. coli* was around 1 · 10^−6^–1 · 10^−5^ μm^2^ s^−1^. According to Nichols (2017), who proposed a “rule of thumb” to classify chemical and biochemical compounds according to membrane permeability, impermeant was characterized by permeability coefficients lower than 1 · 10^−10^ cm s^−1^ (or *D* < 5 · 10^−8^ μm^2^ s^−1^), while permeants were considered with permeability coefficients higher than 1 · 10^−8^ cm s^−1^ (or *D* > 5 · 10^−6^ μm^2^ s^−1^). Therefore, when the *in-silico* modeling undertaken in this study were considered (Fig. 2A and B), IPTG may be considered a permeant to the cytoplasmic membrane, with *D* of approximately 1 · 10^−6^ μm^2^ s^−1^. Retrospective data obtained from the literature on diffusion of small molecules and proteins in diluted aqueous solution was next compared. A log–log correlation between molecular weight and diffusion coefficient has been documented for a wide range of molecular masses at a given temperature, as demonstrated in Supplementary Material 10.4 (Potma et al., 2001; Stewart, 2003; Hahn & Aragon, 2006; Ribeiro et al., 2006; Di Cagno et al., 2018). Based on these retrospective data and interpolation for IPTG molecular mass, the diffusion coefficient of IPTG in aqueous solutions at 23–25°C was estimated to be approximately 500 μm^2^ s^−1^ (Supplementary Material 10.4).

The diffusion of a small molecule in a diluted aqueous solution may be considered the upper limit for the diffusion coefficient. To get a better estimation for IPTG uptake by *E. coli*, it was important to evaluate the diffusion in other environments, such as synthetic lipid membranes or cytoplasmic membrane of *E. coli*. However, data in the literature was scarce since the quantification and prediction of diffusion coefficients in complex systems is challenging. Moreover, several variables are known to affect the diffusion coefficient, such as the concentration of components in the solution, temperature, osmolality, permeant net charge, and permeant dipole moment (Schavemaker et al., 2018; Wilhelm et al., 2019).

Despite the widespread use of IPTG as an inducer in heterologous protein expression in *E. coli* for many decades, data on diffusion of IPTG or other galactosides across lipid membranes are surprisingly unknown. One related example is the diffusion of three antibiotics (tetracycline and two derivatives) across a synthetic *E. coli* membrane (Sigler et al., 2000). These experimental data indicated that the diffusion coefficients of the two permeant compounds (tetracycline and DMG-DMDOT) were of the same order of magnitude as the estimation for IPTG simulated herein (Item 4.1), while the diffusion coefficient of the impermeant compound (2-tetracyclinonitrile) was three orders of magnitude lower (Supplementary Material 10.5).

The *in silico* simulation indicated that diffusion of IPTG across the cytoplasmic membrane of *E. coli* was a relevant process when considering the rate of IPTG uptake and the intracellular IPTG concentration, and this was in agreement with experimental reports of others (Winkler & Wilson, 1966; Tran et al., 2015). In the absence of specific carriers, galactosides with *D* greater than 1 · 10^−6^ μm^2^ s^−1^ had an equilibration time of less than 1 hr when the difference in IPTG concentration was 1 mM. The relative contribution of diffusion in the presence of carriers is discussed in the subsection Combined Transport.

### Active Transport

Initial OVAT based simulations were performed to evaluate the individual contributions the main variables *N*_lacY_, *k*_cat_, *K*_M_, and ${\bar{V}}_{{\mathrm{cell}}}$ provided for IPTG transport (Fig. 4A–D). This was followed by a sensitivity analysis test to compare the relative influence of each variable (Fig. 5A and B). In all cases, the initial external IPTG concentration was 1 mM. Even though diffusion time is an unusual nomenclature to be used, in this case it was preferred to quantify the necessary time for the internal IPTG concentration to equalize with that of the external concentration in such a way that the results from simple diffusion and active transport simulations might be compared. Following attainment of equilibrium, the intracellular concentration of IPTG was found to reach a limit of approximately 100 mM, as shown in Fig. 4A–D. This concentration level corresponded to a concentration factor of 100, which was consistent with previous findings reported in the literature (Rickenberg et al., 1956; Novick and Weiner, 1957; Robertson et al., 1980). The sensitivity analysis indicated that, in the range reported in literature for all variables, ${\bar{V}}_{{\mathrm{cell}}}$ was least influential followed by *K*_M_ and then *k*_cat_. The most influential variable on IPTG active transport was *N*_lacY_ (Fig. 5B). In the context of the model presented in this study, cell volume represented a crucial factor that must be considered as this had a direct impact on the calculation of the rate of IPTG uptake by simple diffusion or active transport (Equations 7–10). The size of *E. coli* cells is also highly influenced by cultivation conditions. A significant correlation has been established between the average cell volume and the growth rate (Schaechter et al., 1958), which is contingent upon various factors such as temperature, nutrient availability and metabolite concentration (Volkmer & Heinemann, 2011; Iyer-Biswas et al., 2014; Taheri-Araghi et al., 2015; Ojkic et al., 2019). For instance, an investigation of cultivation conditions that resulted in growth rates ranging from 0.84 to 2.54 h^−1^ revealed that the average cell volume of newly generated cells was in the range of 0.44–2.77 μm^3^. As a reference value for the simulations presented in this study, experimental data from Taheri-Araghi et al. (2015) was used. In this case, for cells that grew on medium containing glucose and 12 amino acids at a rate of 1.605 ± 0.496 h^−1^, the average volume of new cells was 1.044 ± 0.153 μm^3^ (Fig. 1). These results demonstrated that amongst the variables studied, ${\bar{V}}_{{\mathrm{cell}}}$ had the least effect on the rate of IPTG uptake by active transport (Figs. 4D and 5B). This was primarily due to limited variation of this parameter under physiological conditions. Strict regulation of cell dimensions, particularly the ratio between surface area and volume (Harris and Theriot, 2018; Ojkic et al., 2019; Shi et al., 2021), is a fundamental aspect for maintaining physiological functions, including nutrient uptake and metabolic product excretion (Koch, 1990). Additionally, changes in cell volume are dependent upon cell division time, making it a slow process compared to the rapid expression of new proteins, such as the expression of lacY.

The *in silico* simulations suggested that when *K*_M_ values were below 100 μM, the equilibration time was less than one hour (Fig. 4C). Further reductions of *K*_M_ values did not result in a decrease in the equilibration time, in accordance with predictions of a Michaelis–Menten type equation (Equation 9) used herein to model the rate of active transport. The equation predicted that the maximum rate was attained at sufficiently low *K*_M_ values. The *in silico* simulations predicted that 100 μM corresponded to 10% of the initial extracellular IPTG concentration of 1 mM. Experimental data of *K*_M_ for several substrates of the lactose permease were mostly in the range between 100 and 1000 μM (Kepes, 1960; Winkler & Wilson, 1966; Maloney & Hastings Wilson, 1973; Lancaster et al., 1975; Huber et al., 1980; Robertson et al., 1980; Viitanen et al., 1984; Lolkema et al., 1991; Ozbudak et al., 2004; Dekel & Alon, 2005), which suggested that for an extracellular IPTG concentration of 1 mM, any change in the *K*_M_ value greatly influenced the IPTG uptake rate and, consequently, the equilibration time. Therefore, this property could be a useful parameter for future optimization, in order to modulate the equilibration time. For instance, one could generate lacY mutants to increase *K*_M_ or reduce the external IPTG concentration well below the *K*_M_, thereby reducing the uptake rate, which has already been archived in several other studies that typically used IPTG at concentrations below 60 μM (Vilar et al., 2003; Marbach & Bettenbrock, 2012; Faust et al., 2015).

The effect of *k*_cat_ was next analyzed. The value range used in the *in silico* simulations (1–30 s^−1^) was chosen based upon several experimental values reported in literature (Robertson et al., 1980; Viitanen et al., 1984; Wright & Overath, 1984; Smirnova et al., 2014). For instance, Viitanen et al. (1984) reported a *k*_cat_ of 16–21 s^−1^ for lactose transport by lacY when reconstituted in proteo-liposomes. This value agreed with data from Robertson et al. (1980) and Wright *and* Overath (1984) who both reported a *k*_cat_value of 4.3 s^−1^ for lactose transport by lacY reconstituted in phospholipid vesicles; and Smirnova et al. (2011) who employed spectrofluorometry to more precisely determine a *k*_cat_value of 21 ± 3.6 s^−1^ for NPG transport by wild type lacY also in proteoliposomes. The transport of IPTG from the periplasm to the cytoplasm presents a multi-step mechanism, the rate limiting step of which is the opening of a periplasmic cavity for substrate binding, which is independent of the substrate concentration (Smirnova et al., 2011, 2014). Since the primary amino acid sequence of lacY is highly conserved, changes to the lacY sequence were not expected in commercially available strains of *E. coli* and, no variation in the turnover number was expected. Therefore, for further simulations a reference number for *k*_cat_ of 20 s^−1^ was used.

Results presented herein indicated that the greatest influence upon the rate of IPTG uptake was, not surprisingly, the number of lacY proteins per cell (Figs. 4A and 5B). The number of copies of most proteins in a population of *E. coli* cells is known to follow a gamma distribution with a rather broad range, due to the stochastic nature of the regulation of gene expression (Li and Xie, 2011). In a given experiment for quantification of more than 1000 tagged proteins, about 50% of these were expressed at an average level of less than 10 copies per cell (Li and Xie, 2011), which agreed with data presented elsewhere (Taniguchi et al., 2010; Wiśniewski & Rakus, 2014). A careful analysis of these available data suggested that there is an upper limit in the order of 10^4^ copies of any protein in a cell and, that the average copy numbers tend to be in the lower range. For these reasons, a range between 10 and 1000 molecules per squared micrometer of cell surface, which for a volume of 1.044 μm^3^ (≈ 6.5 μm^2^) corresponded to around 65 and 6500 lacY copies per cell was chosen in this study. For this range, a wide variation in *I*_int_ and equilibration time were observed from the *in-silico* simulated modelling (Fig. 4A). Rapid equilibration times of less than 2 hr were only observed in simulations where more than 500 copies·μm^−2^ were modeled. Interestingly, it is known that low copy numbers of lacY per cell are insufficient to trigger induction and, there is a threshold in the intracellular concentration of IPTG necessary to irreversibly change cell phenotype (Novick & Weiner, 1957; Cohn & Horibata, 1959; Choi et al., 2006). For example, Choi et al. (2008) reported that less than 10 lacY molecules per cell were insufficient to trigger induction, and that between 200 and 800 lacY molecules were necessary to irreversibly change cell phenotype. Conversely, (Cohn & Horibata 1959) estimated that 200 ± 100 molecules per cell were sufficient to fully induce protein expression in *E. coli* cells. Other factors are also related to the level of gene expression, such as allosteric regulation of transcription (Monod et al., 1965; Razo-Mejia et al., 2018), but this was not the focus of the modeling reported here. Despite not specifically investigating expression of recombinant proteins, the predicted results from the *in-silico* simulations suggested that the number of lactose permeases was a critical factor in regulating intracellular IPTG concentration and, the expression rate of proteins regulated by the lac operon. This was in agreement with previous observation of others, that cells exhibit rapid upregulation of lactose permeases resulting in a prompt transition from an repressed to induced state (Pardee et al., 1959).

### Combined Transport

The combined effect of simple diffusion and active transport of IPTG across the cytoplasmic membrane was first evaluated for a single condition (Fig. 6A–C). Within the first minute of the simulation, *I*_int_ surpassed *I*_ext_ and reached a plateau after around 40 min, when diffusion and active transport rates equalized. This profile was expected to be the case for any constant number of lacY molecules per cell. This was because as the IPTG transport rate remained approximately constant, the intracellular accumulation of IPTG triggered its own efflux via simple diffusion until the IPTG influx and efflux rates equalized, ultimately reaching an equilibrium state (Fig. 8B). However, the contribution of simple diffusion was more pronounced in cells with small numbers of lacY molecules. For example, in Fig. 7A, where 10 copies of lactose permease per μm^−2^ were considered, the contribution of diffusion for the initial uptake rate was high (54.6 % of the total rate), but this plummeted to 1.2% when there were 1000 lacY molecules per μm^−2^. Figure 7B demonstrated that the impact of diffusion was more perceptible when the value of *I*_int_ at equilibrium was evaluated and, was only evident when lacY was at high copy numbers approaching the limit of *I*_int_ at equilibrium (>5000 molecules·μm^−2^), which was not expected to occur under physiological conditions (Li & Xie, 2011; Taniguchi et al., 2010; Wiśniewski & Rakus, 2014). Therefore, when intermediate numbers of lacY are considered that are more typical under physiological scenarios, the value of *I*_int_ at equilibrium was significantly associated with the number of lacY copies.

To provide a broader analysis of the *I*_int_ kinetic profile in the presence or absence of simple diffusion, a detailed investigation when varying *N*_lacY_ values was conducted (Figs. 8A–B and 9A–B). The kinetic profile of *I_int_* without simple diffusion (Fig. 8A) provided a constant limit of 100 mM, which was 100 times the *I_int_* value, for the simulated conditions, whereas higher numbers of lacY led to faster equilibration. However, when simple diffusion was considered, the relationship between *N*_lacY_ and *I*_int_ became clear (Fig. 8B). The observed kinetic profile resembled that reported by Maloney & Hastings Wilson, (1973), where *I*_int_ was correlated with the number of transporters, whereas the equilibration time was not (Fig. 8C). A second set of simulations was conducted to evaluate the combined impact of active transport and diffusion on the internal concentration of IPTG, under conditions whereby the external concentration of IPTG was varied whilst the number of transporters was kept constant (Fig. 9A and B). In both situations, there was a direct correlation between *I*_int_ and *I*_ext_ at equilibrium. However, when diffusion was absent (Fig. 9A), the concentration of intracellular IPTG was 100 times higher than the external concentration, while the presence of diffusion led to a lower internal concentration of IPTG at equilibrium due to concentration-driven counter flux. For example, given a constant number of permeases of 200 molecules·μm^−2^, *I*_int_ at equilibrium was around 100 times *I*_ext_ when simple diffusion was not considered, but around 30 times when a diffusion coefficient of 1 · 10^−6^ μm^2^·s^−1^ was taken into account (Fig. 8A and B). The relationship between *I*_int_ at equilibrium and *I*_ext_ (Fig. 9A and B) allowed for the precise adjustment of *I*_ext_ to achieve a desired *I*_int_ at equilibrium. For instance, at a constant number of 200 permeases·μm^−2^, *I*_int_ at equilibrium was 100 times greater than *I*_ext_ in the absence of simple diffusion, and 30 times greater than *I*_ext_ when simple diffusion was considered. However, the permease gene is encoded as a chromosomal copy of the *lac* operon in *E. coli* (Jacob et al., 1964) and even small amounts of IPTG can trigger expression of more permeases, which in turn increase the uptake rate of IPTG (Novick & Weiner, 1957). A strategy to modulate *I*_int_ by adjusting *I*_ext_ would only be effective if expression of lacY were no longer regulated by lacI. In this case, the number of lacY per cell should be determined in another way as, for example, by placing the lacY gene under the regulation of an alternative promoter and modifying the ribosome binding site to regulate the copy number of the protein (Salis et al., 2009; Garcia & Phillips, 2011; Brewster et al., 2012).

### Limitations of the Model and Opportunities for Future Applications

While the simplicity of the model described herein limits evaluation of variables such as cell or protein number, or cell volume; these simplifications do facilitate easier interpretation of results and, allow these results to be directly compared to data from existing literature. The model does allow for confounding factors to be eliminated, such as the effect of dilution (e.g. substrate feed or cell growth) on inducer concentration. This approach provides fundamental questions to be explored, including the extent of simple diffusion and influence of cell volume on the control of inducer uptake, and thereby, protein expression. As demonstrated in this research, the model has proven useful in estimating the diffusion coefficient of IPTG across the *E. coli* cytoplasmic membrane and, evaluating the significance of lacY kinetic parameters in IPTG uptake. It also offers a valuable biophysical approach to modeling inducer uptake and has the potential for broader applications, including direct integration into existing models for recombinant protein expression. Importantly, the mass balance equations can be easily adapted to accommodate variable lacY numbers, higher cell densities and cell growth. Additionally, the model is suitable for stochastic investigations, such as assessing the effects of cell volume distribution and protein number distribution.

## Conclusion

An *in-silico* model was developed to estimate the quantitative contribution of simple and active uptake of IPTG into *E. coli* cells. Such analytical and numerical understanding of the diffusion mechanism was important to fill a surprising knowledge gap to better control recombinant protein expression. Contrary to accepted thinking, the modeling described herein indicated that implementing culture conditions that controlled cell volume were unlikely to be a successful strategy to enhance IPTG mediated expression of recombinant proteins under lac operon control, since cell volume had insignificant impact on IPTG uptake. The parameter that most influenced simple diffusion was the diffusion coefficient, while the number of enzymes per area was the most important parameter affecting active transport. As anticipated, active transport outperforms diffusion in terms of the velocity of IPTG uptake. However, the wild type lacY appears unsuitable for regulating the intracellular IPTG concentration and achieving tunable expression of recombinant proteins in *E. coli*, which is known to increase protein yield. Optimizing *k*_cat_ and *K*_M_ parameters by targeted mutation of the galactoside binding site in lacY could be a future strategy to improve the performance of recombinant protein expression. For example, if *k*_cat_ were reduced while *K*_M_ was increased, active transport of IPTG across the cytoplasmic membrane would be reduced, thereby lessening the metabolic burden on the cell and expediating accumulation of recombinant protein. The computational model described herein is made freely available and is amenable to optimize recombinant protein expression in other heterologous hosts.

## Supplementary Material

kuad034_Supplemental_File
