# Supplementary material for: Mechanistic aspects of IPTG (isopropylthio-β-galactoside) transport across the cytoplasmic membrane of Escherichia coli—a rate limiting step in the induction of recombinant protein expression
Source: J Ind Microbiol Biotechnol. 2023 Oct 17;50(1):kuad034. doi: 10.1093/jimb/kuad034 (PMC10639102; doi:10.1093/jimb/kuad034)
Supplement: kuad034_Supplemental_File [file kuad034_supplemental_file.docx]

$\frac{1}{P}=\frac{1}{P_{1}}+\frac{1}{P_{2}}+\ldots+\frac{1}{P_{n}}$ $P_{m}=\frac{KD}{W_{m}}$ $S=2\pi V^{2/3}$ $\bar{V}_{cell}$ $\rho_{cell}$ $\frac{dI_{int}}{dt}=\frac{r_{total}}{\bar{V}_{cell}}\cdot\left| {10}^{15} \right|$ $\frac{dI_{ext}}{dt}=-\frac{r_{total}\cdot X}{\bar{V}_{cell}\cdot\rho_{cell}}\cdot\left| {10}^{15} \right|$ $r_{total}$ $r_{diff}$ $r_{act}$ $r_{total}= r_{diff}+r_{act}$ $r_{diff}$ $r_{diff}$ $D$ $\bar{S}_{cell}$ $I_{ext}$ $I_{int}$ $W$ $r_{diff}=\frac{D\cdot\bar{S}_{cell}}{W}\cdot\left( I_{ext}-I_{int} \right)\cdot\left| {10}^{-15} \right|$ $\bar{S}_{cell}=2\pi{\bar{V}_{cell}}^{2/3}$ $r_{act}$ $I_{ext}$ $k_{cat}$ $K_{M}$ $lacY$ $N_{lacY}$ $\bar{S}_{cell}$ $N_{A}$ $v_{max}$ $r_{act}=\frac{lacY\cdot k_{cat}\cdot I_{ext}}{K_{M}+I_{ext}}$ $lacY=\frac{N_{lacY}\cdot\bar{S}_{cell}}{N_{A}}$ $v_{max}=\frac{lacY*k_{cat}}{\bar{V}_{cell}}*\left| 6.0\cdot{10}^{19} \right|$ $t_{d}$ $I_{int}$ $I_{ext}$ $\theta_{d}$ $I_{int}$ $\theta_{d}$ $N_{lacY}$ $k_{cat}$ $K_{M}$ $\bar{V}_{cell}$ $N_{lacY}$ $k_{cat}$ $K_{M}$ $\bar{V}_{cell}$ $X$ $\rho_{cell}$ $D$ $W$ $k_{cat}$ $K_{M}$ $K_{M}$ $N_{lacY}$ $k_{cat}$ $K_{M}$ $\bar{V}_{cell}$ $N_{lacY}$ $k_{cat}$ $K_{M}$ $\bar{V}_{cell}$ $N_{lacY}$ $N_{lacY}$ $k_{cat}$ $K_{M}$ $\bar{V}_{cell}$ $lacY\cdot k_{cat}$ $N_{lacY}$ $N_{lacY}$ $N_{lacY}$ $r=k\cdot{IPTG}_{int}$ $D$ $D$ $D$ $N_{lacY}$ $k_{cat}$ $K_{M}$ $\bar{V}_{cell}$ $\bar{V}_{cell}$ $K_{M}$ $k_{cat}$ $N_{lacY}$ $\bar{V}_{cell}$ $k_{cat}$ $k_{cat}$ $k_{cat}$ $k_{cat}$ $k_{cat}$ $I_{int}$ $I_{int}$ $N_{lacY}$ $I_{int}$ $I_{int}$ $I_{int}$ $I_{ext}$ $N_{lacY}$ $k_{cat}$ $K_{M}$ $\bar{V}_{cell}$ $N_{lacY}$ $k_{cat}$ $K_{M}$ $\bar{V}_{cell}$ $N_{lacY}$ $k_{cat}$ $K_{M}$ $\bar{V}_{cell}$ $N_{lacY}$ $k_{cat}$ $K_{M}$ $\bar{V}_{cell}$ $N_{lacY}$Supplementary material

## Calculation report – Simple Diffusion Rate

**Considerations**

- Diffusion coefficient is constant;
- Average cell volume is constant;
- Average membrane width is constant;
- Membrane was considered a flat surface.

**Modelling**

Diffusion is the movement of molecules through a fluid driven by a concentration gradient. Due to the random nature of the migration, molecules move at high speeds, but travel only small distances, resulting in a slow net velocity. Although the trajectory is random, the step-size is not random and might be captured by one single parameter, called diffusion coefficient (Nagy, 2012; Schavemaker et al., 2018). The Fick’s law correlates the diffusion coefficient ($D$), the concentration gradient across a fluid layer and the molecule transfer rate according to Equation S1, where $J$ is the transfer rate, $I$ is the permeant concentration, or IPTG (inducer) in our case, and $x$ is the space coordinate in the direction of diffusion.

$J=D\frac{dI}{dx}$ (Eq. S1)

Considering the diffusion across the cytoplasmic membrane with width W, intracellular IPTG concentration $I_{\mathrm{int}}$, and extracellular IPTG concentration $I_{\mathrm{ext}}$, Equation S1 is integrated, as follows:

$\int_{0}^{W} Jdx=-D\int_{I_{ext}}^{I_{int}} dI$ (Eq. S2)

$J\cdot W=-D\cdot\left( I_{int}-I_{ext} \right)$ (Eq. S3)

$J=\frac{D}{W}\cdot\left( I_{ext}-I_{int} \right)$ (Eq. S4)

Simple diffusion rate ($r_{\mathrm{diff}}$) is a function of $J$ and membrane area, or average cell surface area ($\bar{S}_{\mathrm{cell}}$ ), according to Equation S5.

$r_{diff}=J\cdot\bar{S}_{cell}$ (Eq. S5)

Considering that the cell membrane may be approximated as a flat sheet membrane, then, Equations S4 and S5 are combined and become Equation S6.

$r_{diff}=J\cdot\bar{S}_{cell}=\frac{D\cdot\bar{S}_{cell}}{W}\cdot\left( I_{ext}-I_{int} \right)$ (Eq. S6)

Equation S6 was converted into Equation S7 to adjust the units of the input variables $I_{\mathrm{ext}}$ and $I_{\mathrm{int}}$ to mol L^-1^.

$r_{diff}=J\cdot\bar{S}_{cell}=\frac{D\cdot\bar{S}_{cell}}{W}\cdot\left( I_{ext}-I_{int} \right)\cdot{10}^{-15}$ (Eq. S7)

Where

| Variable | Description | Unit |
| --- | --- | --- |
| $\boldsymbol{I}_{\boldsymbol{ext}}$ | Extracelullar IPTG concentration | mol μm^-3^ |
| $\boldsymbol{I}_{\boldsymbol{int}}$ | Intracellular IPTG concentration | mol μm^-3^ |
| $\boldsymbol{D}$ | Diffusion coefficient of IPTG across cell membrane | μm^2^ s^-1^ |
| $\boldsymbol{J}$ | Transfer rate (IPTG flux across the membrane) | mol s^-1^ μm^-2^ |
| $\boldsymbol{r}_{\boldsymbol{diff}}$ | IPTG uptake rate by simple diffusion | mol s^-1^ cell^-1^ |
| ${\bar{\boldsymbol{S}}}_{\boldsymbol{cell}}$ | Average surface area of one cell | µm^2^ |
| $\boldsymbol{W}$ | Average width of cytoplasmic membrane | μm |

## Calculation report – Active transport

Active transport of IPTG across the cytoplasmic cell membrane of *E. coli* via lacY follows the Michalis-Menten equation (Rickenberg et al., 1956), according to Eq. S8. lacY is the quantity of lactose permease molecules per cell, which is calculated from the average number of lactose permeases per area, the average cell surface area, and the Avogadro number, according to Eq. 9.

$r_{act}=\frac{lacY\cdot k_{cat}\cdot I_{ext}}{K_{M,lacY}+I_{ext}}$ (Eq. S8)

$lacY=\frac{N_{lacY}\cdot\bar{S}_{cell}}{N_{A}}$ (Eq. S9)

To compare our simulated data with experimental ones, the maximum enzyme velocity of active transported was calculated (Equation S10)

$v_{max}=\frac{lacY*k_{cat}}{\bar{V}_{cell}}*\left[ 6,0\cdot{10}^{19}\frac{\mu mol}{mol}\frac{{\mu m}^{3}}{{cm}^{3}}\frac{s}{min} \right]$ (Eq. S10)

| Variable | Description | Unit |
| --- | --- | --- |
| $\boldsymbol{I}_{\boldsymbol{ext}}$ | Extracelullar IPTG concentration | mol μm^-3^ |
| $\boldsymbol{k}_{\boldsymbol{cat}}$ | Turnover number | s^-1^ |
| $\boldsymbol{K}_{\boldsymbol{M}}$ | Michaelis constant for IPTG uptake by lacY | mol L^-1^ |
| $\boldsymbol{lacY}$ | Quantity of lactose permease per cell | mol cell^-1^ |
| $\boldsymbol{N}_{\boldsymbol{lacY}}$ | Lactose permease copy number per cell surface area | molecules µm^-2^ |
| $\boldsymbol{r}_{\boldsymbol{act}}$ | IPTG uptake rate by active transport | mol s^-1^ cell^-1^ |
| ${\bar{\boldsymbol{V}}}_{\boldsymbol{cell}}$ | Average volume of one cell | µm^3^ |
| $\boldsymbol{v}_{\boldsymbol{max}}$ | Maximum velocity of uptake per cell volume | μmol min^-1^ cm^-3^ |

## Calculation report – Mass balance

**Considerations**

- Average cell volume is constant;
- Total volume of cells is irrelevant compared to medium volume;
  - $(N_{\mathrm{cells}}\cdot\bar{V}_{\mathrm{cell}})\ll V_{\mathrm{medium}}$
- Number of cells is constant. Cells are not dividing.
- Medium volume is constant.

**Modelling**

The general mass balance of a given compartment is given by Equation S11

$\frac{dM}{dt}=Q_{in}-Q_{out}+Q_{produced}-Q_{consumed}$ (Eq. S11)

Where $\frac{\mathrm{dM}}{\mathrm{dt}}$ is the variation of mass in a time fraction $\mathrm{dt}$, and $Q_{x}$ is the transport rate in mol s^-1^. For mathematical purpose, the inducer (IPTG) transport rate was assumed to be positive when IPTG enters the cell.

*Mass balance of intracellular IPTG concentration*

The variation of IPTG quantity inside the cells ($M_{\mathrm{int}}$) is proportional to the total mass transport rate ($Q_{\mathrm{total}}$), according to Equation S12.

$\frac{dM_{int}}{dt}=Q_{total}$ (Eq. S12)

$Q_{\mathrm{total}}$ may be expressed as the total IPTG transport rate per cell ($r_{\mathrm{total}}$) multiplied by the number of cells ($N_{\mathrm{cells}})$. The IPTG quantity inside the cells may be decomposed into the number of cells, the average volume of one cell ($\bar{V}_{\mathrm{cell}}$) and the IPTG concentration inside the cells ($I_{\mathrm{int}}$) (Equation S13).

$\frac{d(N_{cells}\cdot\bar{V}_{cell}\cdot I_{int})}{dt}=r_{total}\cdot N_{cells}$ (Eq. S13)

Considering that the cells are not dividing, and the average cell volume is constant, Equation S13 becomes Equation S14.

$N_{cells}\cdot\bar{V}_{cell}\frac{dI_{int}}{dt}=r_{total}\cdot N_{cells}$ (Eq. S14)

Simplifying Equation S14:

$\bar{V}_{cell}\frac{dI_{int}}{dt}=r_{total}$ (Eq. S15)

Rearranging, Equation S15 becomes Equation S16.

$\frac{dI_{int}}{dt}=\frac{r_{total}}{\bar{V}_{cell}}$ (Eq. S16)

To adjust the units, Equation S16 becomes Equation S17.

$\frac{dI_{int}}{dt}=\frac{r_{total}}{\bar{V}_{cell}}\cdot\left| \frac{{10}^{15}{\mu m}^{3}}{L} \right|$ (Eq. S17)

*Mass balance of extracellular IPTG concentration*

The variation of IPTG quantity in the medium ($M_{\mathrm{ext}}$) is proportional to the total mass transport rate ($Q_{\mathrm{total}}$) (Equation S18).

$\frac{dM_{ext}}{dt}=-Q_{total}$ (Eq. S18)

$Q_{total}$ may be expressed as the total IPTG transport rate per cell ($r_{\mathrm{total}}$) multiplied by the number of cells ($N_{\mathrm{cells}})$. The IPTG quantity in the medium may be decomposed into the volume of medium ($V_{\mathrm{medium}}$) and the IPTG concentration in the medium ($I_{\mathrm{ext}}$) (Equation S19).

$\frac{d(V_{medium} I_{ext})}{dt}=-r_{total}\cdot N_{cells}$ (Eq. S19)

Considering that the volume occupied by cells is much smaller than the volume of medium, and the cells are not dividing, the volume of medium is considered constant. Hence, Equation S19 becomes Equation S20.

$V_{medium}\frac{dI_{ext}}{dt}=-r_{total}\cdot N_{cells}$ (Eq. S20)

Rearranging Equation S21, the ODE for extracellular IPTG concentration is obtained (Equation S21)

$\frac{dI_{ext}}{dt}=-\frac{r_{total}\cdot N_{cells}}{V_{medium}}$ (Eq. S21)

Considering that the volume of cells is negligible compared to the volume of medium,

$N_{\mathrm{cells}}$ may be calculated from the values of cell concentration ($X$), $V_{\mathrm{medium}}$, cell density ($\rho_{\mathrm{cell}}$), $\bar{V}_{\mathrm{cell}}$, and a factor to convert units, according to Equation S22.

$N_{cells}=\frac{X\cdot V_{medium}}{\rho_{cell}\cdot\bar{V}_{cell}}\cdot\left| \frac{{10}^{15}{\mu m}^{3}}{L} \right|$ (Eq. S22)

Substituting Eq. S22 in Eq. S21:

$\frac{dI_{ext}}{dt}=-\frac{r_{total}\cdot X}{\rho_{cell}\cdot\bar{V}_{cell}}\cdot\left| \frac{{10}^{15}{\mu m}^{3}}{L} \right|$ (Eq. S23)

*Combined equations*

In resume, the ordinary differential equations (ODEs) for intracellular and extracellular IPTG concentrations are:

$\frac{dI_{int}}{dt}=\frac{r_{total}}{\bar{V}_{cell}}\cdot\left| {10}^{15} \right|$ (Eq. S24)

$\frac{dI_{ext}}{dt}=-\frac{r_{total}\cdot X}{\bar{V}_{cell}\cdot\rho_{cell}}\cdot\left| {10}^{15} \right|$ (Eq. S25)

Where

| Variable | Description | Unit |
| --- | --- | --- |
| $\boldsymbol{I}_{\boldsymbol{ext}}$ | Extracelullar IPTG concentration | mol L^-1^ |
| $\boldsymbol{I}_{\boldsymbol{int}}$ | Intracellular IPTG concentration | mol L^-1^ |
| $\boldsymbol{N}_{\boldsymbol{cells}}$ | Number of cells | cell |
| $\boldsymbol{r}_{\boldsymbol{total}}$ | Total IPTG transport rate per cell | mol s^-1^ cell^-1^ |
| $\boldsymbol{t}$ | time | s |
| ${\bar{\boldsymbol{V}}}_{\boldsymbol{cell}}$ | Average volume of one cell | µm^3^ |
| $\boldsymbol{V}_{\boldsymbol{medium}}$ | Volume of medium | L |
| $\boldsymbol{X}$ | *E. coli* cell concentration | g L^-1^ |
| $\boldsymbol{\rho}_{\boldsymbol{cell}}$ | Cell density of wet *E. coli* cells | g L^-1^ |

10.4. Diffusion coefficients obtained from the literature for some selected compounds in water at 23 – 25°C. (Caffeine, Calcein, Chloramphenicol, Ketoprofen, Nitrofurantoin, Paracetamol, Penicillin G, Tetracycline, Trimethoprim, Vancomycin, Lactose, Sucrose, Glucose, Fructose, Acetic acid, Lysozyme, β-trypsin, α-chymotrypsin, Alkaline phosphatase, β-galactosidase and GFP)


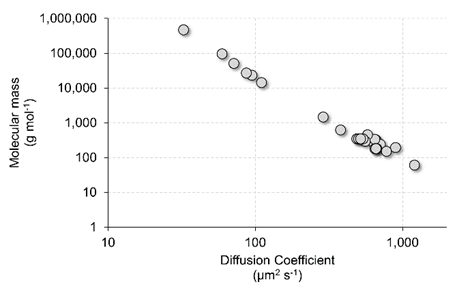


10.5. Experimental data for the diffusion of tetracycline and derivatives across a synthetic *E. coli* membrane (Sigler et al., 2000)**.** Partition coefficient octanol/water, pH 7.8. Diffusion coefficient calculated was based upon the assumption of a membrane width of 50 Å.

| Compound | Molecular mass  (g mol^-1^) | Permeation coefficient  (cm s^-1^) | Partition coefficient octanol/water | Calculated diffusion coefficient  (µm^2^ s^-1^) |
| --- | --- | --- | --- | --- |
| tetracycline | 444.4 | 2.40·10^-9^ | 0.073 | 1.64·10^-6^ |
| 2-tetracyclinonitrile | 426.4 | 6.10·10^-12^ | 0.068 | 4.49·10^-9^ |
| DMG-DMDOT* | 514.5 | 3.30·10^-9^ | 0.077 | 2.14·10^-6^ |

*DMG-DMDOT = 9-(N,N-dimethylglycylamido)-6-demethyl-6-deoxytetracycline
